# A more accurate method acquirement by a comparison of the prediction equations for estimating glomerular filtration rate in Chinese patients with obstructive nephropathy

**DOI:** 10.1186/s12882-016-0345-0

**Published:** 2016-10-18

**Authors:** Meixue Chen, Jumei Xia, Guangchang Pei, Ying Zhang, Shuting Wu, Yushuang Qin, Yuanjun Deng, Shuiming Guo, Yanyan Guo, Gang Xu, Min Han

**Affiliations:** 1 Division of Nephrology, Tongji Hospital, Tongji Medical College, Huazhong University of Science and Technology, 1095 Jiefang Avenue, Wuhan, Hubei 430030 China; 2 Division of nuclear medicine, Tongji Hospital, Tongji Medical College, Huazhong University of Science and Technology, Wuhan, 430030 China

**Keywords:** Obstructive nephropathy, eGFR equation, Glomerular filtration rate

## Abstract

**Background:**

Researchers have developed several equations to predict glomerular filtration rate (GFR) in patients with chronic kidney diseases (CKD). However, there are scarcely any studies performed to discern the best equation to estimate GFR in patients with pure obstructive nephropathy. In present study, we assessed the suitability of six prediction equations and compared their performance in eGFR evaluation for Chinese patients with obstructive nephropathy.

**Methods:**

A total of 245 adult patients with obstructive nephropathy were enrolled. We evaluated the performance of the 3 Modification of Diet in Renal Disease equations (MDRD) (the original MDRD7, 7MDRD; the abbreviated MDRD, aMDRD; and re-expressed abbreviated MDRD, re-aMDRD) and 3 Chronic Kidney Disease Epidemiology Collaboration equations (CKD-EPI) (CKD-EPI equation based on creatinine alone, CKD-EPIcr; CKD-EPI equation based on cystatin C alone, CKD-EPIcys; CKD-EPI equation based on combined creatinine-cystatin, CKD-EPIcr-cys). The measured GFR (mGFR) by ^99^mTc-DTPA renal dynamic imaging method was used as the reference GFR.

**Results:**

The mean age of the study population was 51.61 ± 14.17 and 131 were male (53.47 %). The mean measured GFR was 66.54 ± 23.99 ml/min/1.73 m^2^. Overall, the CKD-EPIcr-cys equation gave the best performance with the best correlation (*R* = 0.72) and agreement (−34.87, 40.83). CKD-EPIcr-cys equation also exhibited the highest accuracy (69.39 %, *P* < 0.01) and diagnostic efficacy (ROC^AUC^ = 0.874) with the smallest bias (2.98, *P* < 0.01). In the subgroup of the lowest GFR, CKD-EPIcys equation exhibited the highest accuracy (52.69 %) and the smallest bias (0.27). In the youngest age subgroup, CKD-EPIcys equation had the highest accuracy (71.64 %) and the smallest bias (−1.24). In other subgroups stratified by GFR, age and gender, CKD-EPIcr-cys equation remained the best performance.

**Conclusion:**

The 3 CKD-EPI equations performed better than the 3 MDRD equations in estimating GFR in Chinese obstructive nephropathy patients; while the CKD-EPI equation based on combined creatinine-cystatin C provided the best estimation of GFR.

## Background

Glomerular filtration rate (GFR) is an important indicator of the filtering capacity of kidneys and is considered the best overall index of renal function currently used [1]. Estimated glomerular filtration rate (eGFR) is the most important variable in the assessment of patients with suspected or known kidney disease in clinical practice [2]. Traditional methods using renal clearances of exogenous inulin, or other alternative exogenous markers (such as iothalamate, EDTA, diethylene triamine pentaacetic acid, and iohexol) can provide accurate GFR evaluation [3]. However, these tests are time consuming and expensive [4], which limits the application to monitor kidney function periodically. As an alternative, GFR estimating equations have been recommended in clinical practice. In 1976, the first creatinine clearance estimating equation - Cockcroft-Gault equation was developed [5]. From then on, researchers have developed and calibrated a series of equations to provide convenient, time-saving and reproducible estimation of kidney function, such as MDRD (Modification of Diet in Renal Disease) and CKD-EPI (Chronic Kidney Disease Epidemiology Collaboration) equations etc. [6–9]. Recently, the CKD-EPI group have developed new equations based on serum cystatin C and combined creatinine-cystatin C, which were shown to perform better than the previous CKD-EPI equation based on serum creatinine alone [10]. However, the new equations have not been externally validated in a Chinese population yet. Obstructive nephropathy refers to the renal disease caused by impaired urine flow or tubular fluid [11], which is taken as one of the most common reasons for chronic kidney disease (CKD) [12–14]. It can be caused by stone, tumor, prostatic hyperplasia, etc. Long-term urinary tract obstruction can lead to renal fibrosis [15]. The pathological process of obstructive nephropathy is different from diffuse renal diseases initiated by immune mechanism, in which the damage of both kidneys are almost the same. However, obstructive nephropathy often occurs unilaterally. Even if it happens bilaterally, the degree of renal damage in the left and right kidneys is not equal. The effects of urinary tract obstruction on renal function must be considered both during and after relief of obstruction and are greatly influenced by whether the obstruction is unilateral or bilateral,acute or chronic, partial or complete [14]. To the best of our knowledge, no studies have validated these eGFR equations only in obstructive nephropathy patients. The current study aimed at testing and comparing the estimations of 6 commonly used eGFR equations (3 MDRD equations and 3 CKD-EPI equations), including the new developed CKD-EPI equation based on combined creatinine-cystatin C [10] in pure obstructive nephropathy patients.

## Methods

### Patients

This study was performed as a retrospective study using resident patients diagnosed with obstructive nephropathy who had underwent a GFR measurement by ^99^mTc-diethylenetriamine penta-acetate (^99^mTc-DTPA) in Tongji Hospital of Huazhong University of Science and Technology (Wuhan, China) between May, 2012, and October, 2013. To be included, the age had to be at least 18 years; the biochemical results, body weight, and height had to be available from the patients’ case records within 3 months of the GFR measurement. For patients who had more than one GFR measurements, the first one was used for analysis. Patients with acute kidney injury, severe edema, pleural effusion or ascites, malnutrition, amputation or skeletal muscle atrophy, heart failure or ketoacidosis were excluded. Patients who were taking cimetidine, trimethoprim or those who were on any kind of renal replacement therapy were also excluded [16]. Finally, a total of 245 individuals were enrolled in the present study.

### GFR measurements

The measurement of GFR was performed using ^99^mTc-DTPA renal dynamic imaging by Ifinia Hawkeye 4 SPECT (GE Healthcare, USA). The identical standard measuring method was as follows: patients were hydrated with 300 ml of water 30mins before the examination. Radioactivity of the syringe containing ^99^mTc-DTPA (provided by Jiangsu Atom Medicine Research Institute, Jiangyuan Pharmaceutical Factory) was counted before injection. And then each of the patients was given a bolus of intravenous injection of approximately 185 MBq DTPA into the forearm. After that, the dynamic renal flow images were collected immediately using the Xeleris^TM^3 Functional Imaging Workstation (GE Healthcare Biosciences, Piscataway, NJ, USA). The post-injection syringe was also counted. Thus the difference of the syringe’s radioactivity between pre- and post-injection was defined as the exact dosage of administered ^99^mTc-DTPA. The calculation of GFR values was done by the Xeleris^TM^3 Functional Imaging Workstation automatically according to the modified Gate’s equation [17, 18]. The measured GFR (mGFR) was standardized by body surface area (BSA) [15].

### Biochemical measurements

Serum creatinine was measured by Roche enzymatic assay (Shanghai Roche Diagnostic Products Co., Ltd, China). Cystatin C was determined by article-enhanced immunoturbidimetry assay (Beijing Leadman Biomedical Co., Ltd, China). All of the fasting blood samples were assayed on a Roche automatic biochemical analyser (cobas 8000 modular analyzer series, Roche Diagnostics Operations, Inc, USA). For patients who had multiple check results, the one nearest date of GFR measurement was used.

### Estimation of GFR

Estimated GFR (eGFR) were calculated by 6 different equations; including 3 MDRD equations (the original MDRD7 [6], the abbreviated MDRD equation [7] and the reexpressed abbreviated MDRD [8] equation, hereafter referred to as the 7MDRD equation, aMDRD equation and re-aMDRD equation respectively); and 3 CKD-EPI equations (CKD-EPI equation based on creatinine alone [9], CKD-EPI equation based on cystatin C alone, and CKD-EPI equation based on combined creatinine-cystatin C [10], hereafter referred to as the CKD-EPIcr equation, CKD-EPIcys equation, and CKD-EPIcr-cys equation respectively). All the equations were all listed in Table 1.

**Table 1:** Equations to estimated GFR

eGFR method	Equation
7MDRD	170 × (Scr)^-0.999^ × (Age^)-0.176^ × 0.762 (if female) × 1.180 (if black) × (BUN^)-0.170^ × (Alb)^0.318^
aMDRD	186 × (Scr)^-1.154^ × (Age)^-0.203^ × 0.742 (if female) × 1.212 (if black)
re-aMDRD	175 × (Scr)^-1.154^ × (Age)^-0.203^ × 0.742 (if female) × 1.212(if black)
CKD-EPIcr	
Female; Scr ≤ 0.7 mg/dl	144 × (Scr/0.7)^−0.329^ × 0.993^Age^ [×1.159 if black]
Female; Scr > 0.7 mg/dl	144 × (Scr/0.7)^−1.209^ × 0.993^Age^ [×1.159 if black]
Male; Scr ≤ 0.9 mg/dl	141 × (Scr/0.9)^−0.411^ × 0.993^Age^ [×1.159 if black]
Male; Scr > 0.9 mg/dl	141 × (Scr/0.9)^−1.209^ × 0.993^Age^ [×1.159 if black]
CKD-EPIcys	
Female or Male; Scys ≤ 0.8 mg/dl	133 × (Scys/0.8)^−0.499^ × 0.996^Age^ [×0.932 if female]
Female or Male; Scys > 0.8 mg/dl	133 × (Scys/0.8)^−1.328^ × 0.996^Age^ [×0.932 if female]
CKD-EPIcr-cys	
Female;	
Scr ≤ 0.7 mg/dl; Scys ≤ 0.8 mg/dl	130 × (Scr/0.7)^−0.248^ × (Scys/0.8)^−0.375^ × 0.995^Age^ [×1.08 if black]
Scr ≤ 0.7 mg/dl; Scys > 0.8 mg/dl	130 × (Scr/0.7)^−0.248^ × (Scys/0.8)^−0.711^ × 0.995^Age^ [×1.08 if black]
Scr > 0.7 mg/dl; Scys ≤ 0.8 mg/dl	130 × (Scr/0.7)^−0.601^ × (Scys/0.8)^−0.375^ × 0.995^Age^ [×1.08 if black]
Scr > 0.7 mg/dl; Scys > 0.8 mg/dl	130 × (Scr/0.7)^−0.601^ × (Scys/0.8)^−0.711^ × 0.995^Age^ [×1.08 if black]
Male;	
Scr ≤ 0.9 mg/dl; Scys ≤ 0.8 mg/dl	135 × (Scr/0.9)^−0.207^ × (Scys/0.8)^−0.375^ × 0.995^Age^ [×1.08 if black]
Scr ≤ 0.9 mg/dl; Scys > 0.8 mg/dl	135 × (Scr/0.9)^−0.207^ × (Scys/0.8)^−0.711^ × 0.995^Age^ [×1.08 if black]
Scr > 0.9 mg/dl; Scys ≤ 0.8 mg/dl	135 × (Scr/0.9)^−0.601^ × (Scys/0.8)^−0.375^ × 0.995^Age^ [×1.08 if black]
Scr > 0.9 mg/dl; Scys > 0.8 mg/dl	135 × (Scr/0.9)^−0.601^ × (Scys/0.8)^−0.711^ × 0.995^Age^ [×1.08 if black]

### Statistical analysis

All statistical analysis were performed in SPSS statistical software, version 19.0 for Windows (SPSS, Chicago, IL, USA). Population characteristics were tested with *t* test, and all data were presented as means ± SD. Body surface area (BSA) was calculated as follows [19]: BSA = (body weight^0.425^[in kg] × height^0.725^[in cm]) × 0.007184. To compare the performance of the equations, Pearson correlation analysis and linear regression were applied to compare the correlation between measured GFR and estimated GFR (Pearson coefficient R was calculated) and bias, precision, and accuracy were also calculated as recommended [20, 21]. Bland-Altman plots were used to study the relation between the GFR and measurement error, we assessed the bias as well as the limits of agreement (calculated as the bias plus or minus 1.96 times of the precision) [22]. We applied area under receiver operating characteristic (ROC^AUC^) curve to describe the diagnostic efficacy of the equations. Paired *t* test and McNemar test were respectively used to test the difference in bias and accuracy between the estimated equations. In addition, bias, precisions and accuracies were also analyzed within the stratifications of GFR, gender, and age, in order to assess the influences of these variables in subgroups. The cutoffs used for GFR stratification were <60, 60 to 90, and >90 ml/min/1.73 m^2^, and for age, it were <45, 45 to 60, and >60 years.

## Results

### Characteristics of the population

In total, 245 Chinese patients with obstructive nephropathy were included in this study, including 131 males and 114 females with a mean age of 51.61 ± 14.17 years. The average value of the measured GFR was 66.54 ± 23.99 ml/min/1.73 m^2^. Detailed laboratory measurements and basic characteristics of the study population were presented in Table 2.

**Table 2:** Characteristics of the study population

Variable	Mean ± SD (*n* = 245) or n[%]
Male gender (n[%])	131(53.5 %)
Age (years)	51.6 ± 14.2
Weight (kg)	62.0 ± 12.1
Height (cm)	163.9 ± 7.2
BMI (kg/m^2^)	23.0 ± 3.6
BSA (m^2^)	1.7 ± 0.2
Serum creatinine (mg/dl)	1.2 ± 1.1
Serum cystatin C (mg/l)	1.4 ± 0.7
Serum urea (mg/dl)	18.0 ± 10.8
Serum albumin (g/dl)	3.7 ± 0.5
Measured GFR (ml/min/1.73 m^2^)	67 ± 24

BMI (kg/m^2^) = body weight (kg) / height (m)^2^

BSA (m^2^) = body weight (kg)^0.425^ × height (cm)^0.725^ × 0.007184

*BMI* body mass index, *BSA* body surface area, *GFR* glomerular filtration rate

### Association and agreement between estimated GFR and measured GFR

**Fig. 1:**
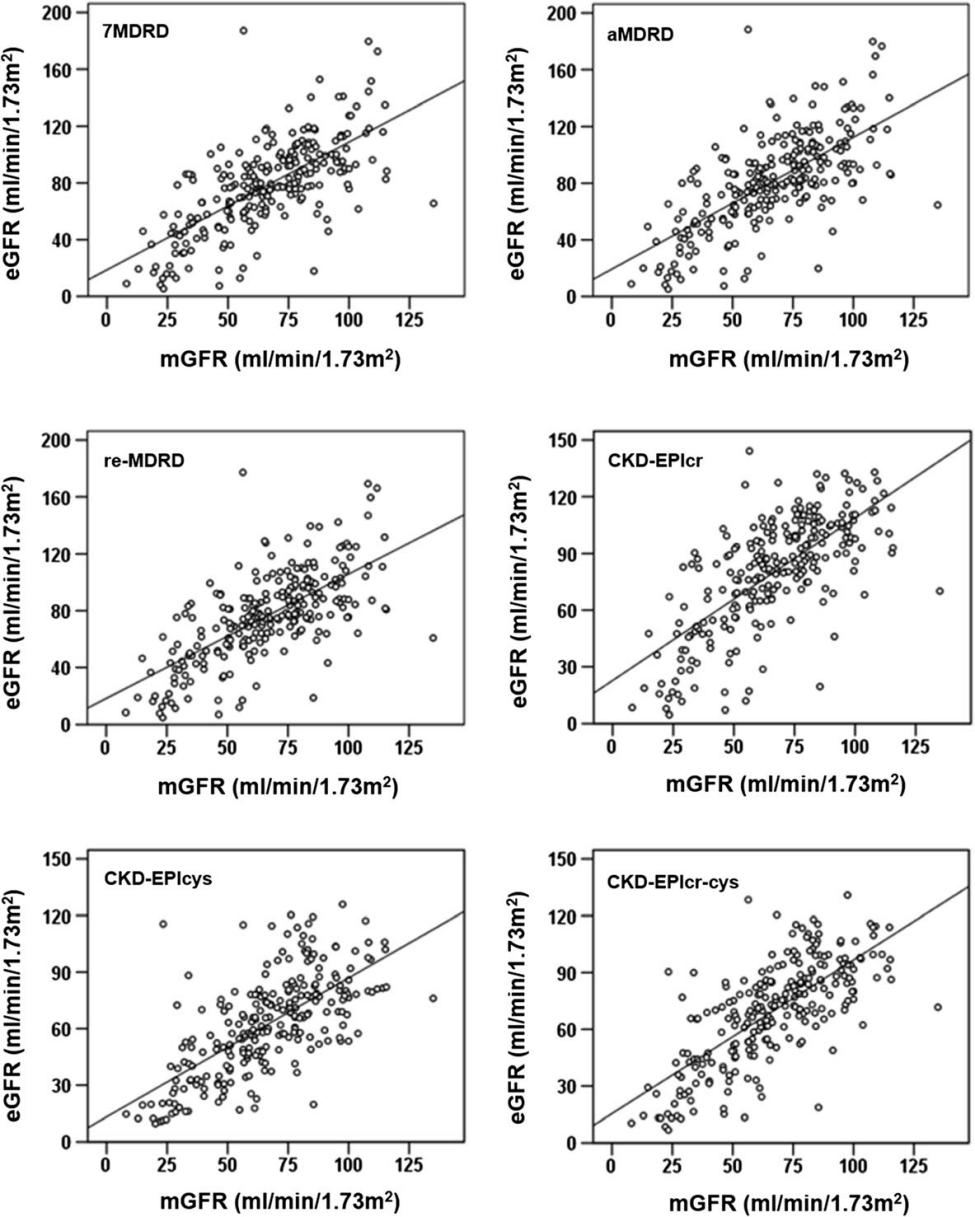
Correlation between measured GFR (mGFR) and estimated GFR (eGFR) using 6 different prediction equations (univariate linear regression model was used, the solid line represents the regression line)

**Fig. 2:**
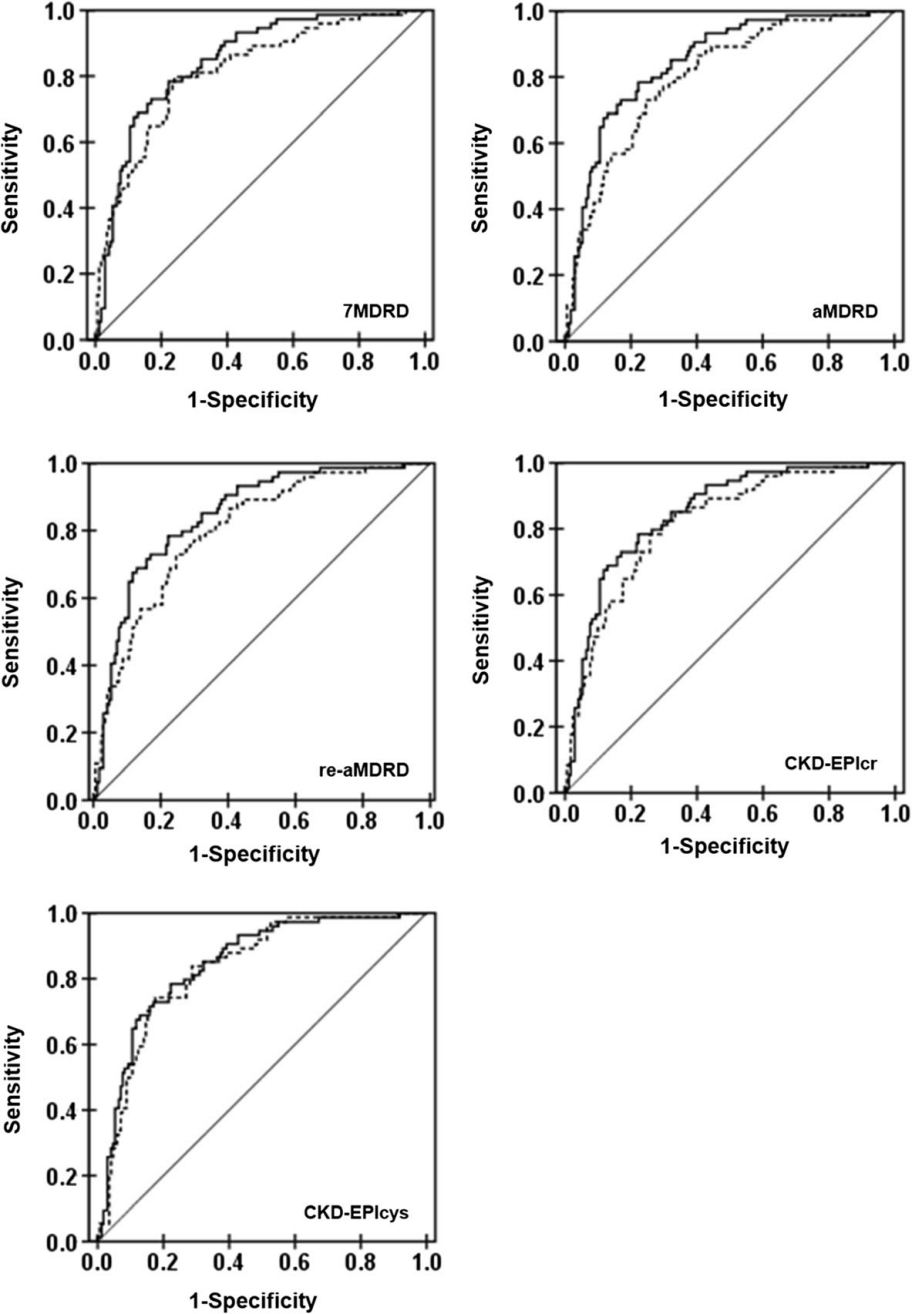
Curves of Receiver Operating Characteristics analysis for the diagnostic ability of CKD-EPIcr-cys equation (solid-line curve) versus other 5 equations (dotted-line curves). The values of the area under the curve were listed in Table 3

**Fig. 3:**
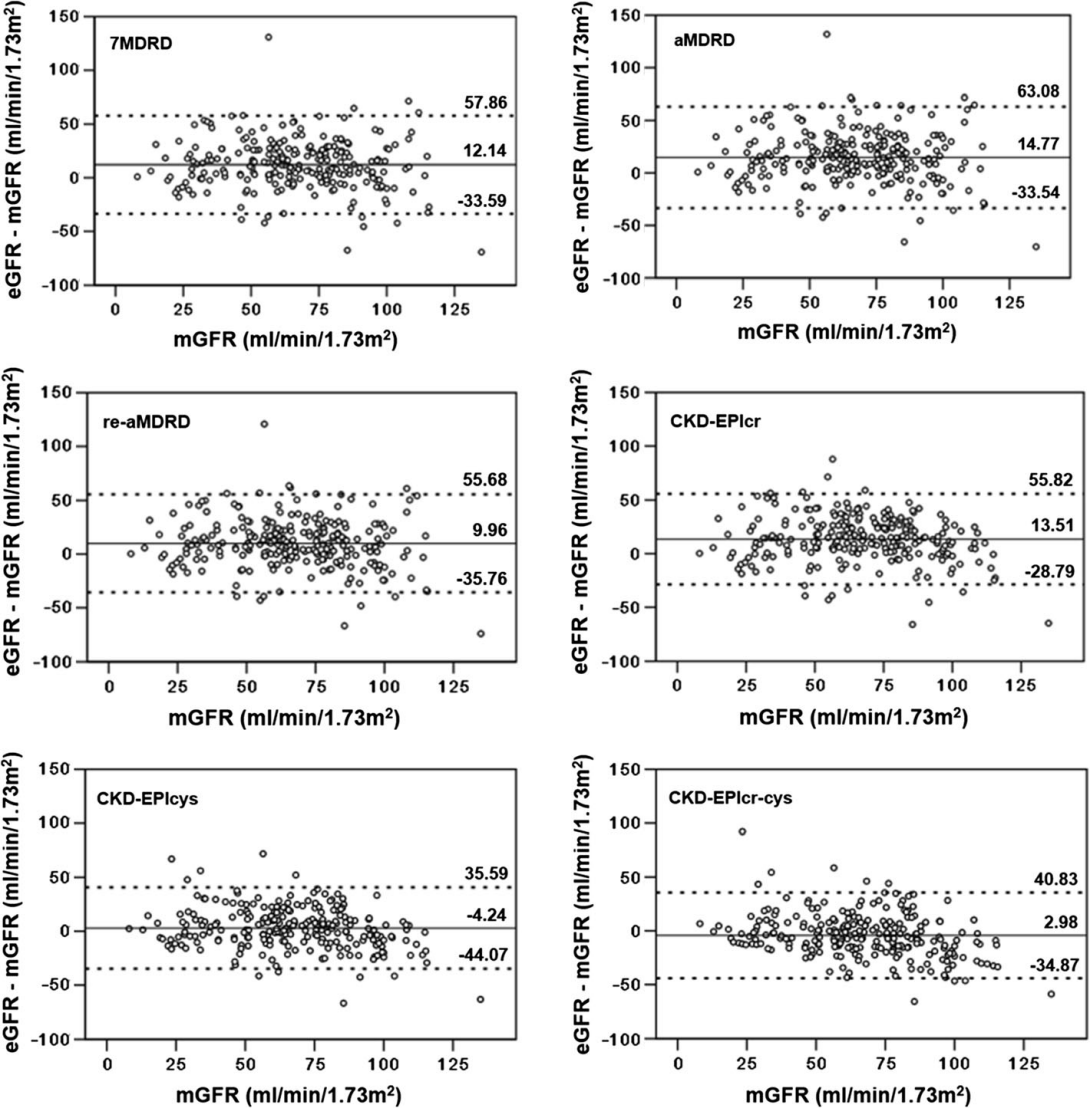
Bland-Altman plots of the estimated and measured GFR. eGFR - mGFR represents the difference between the estimated GFR (eGFR) and the measured GFR (mGFR). A positive difference indicates an overestimation by the equation, whereas a negative difference indicates an underestimation. The solid lines indicate the mean difference; the dashed lines indicate the lines of agreement, calculated as the mean difference ±1.96 SD of this difference

The overall relationships between measured and estimated GFR values were presented in Fig. 1. All of the six prediction equations correlated well with the measured GFR (*p* < 0.001) (Fig. 2). The Pearson correlation coefficients (R) varied from 0.681 to 0.720, and CKD-EPIcr-cys equation showed the best correlation (*R* = 0.720, shown in Table 3). CKD-EPIcr-cys equation also gave the highest accuracy (*P* < 0.01), the smallest bias (*P* < 0.01) (Table 4) and the best diagnostic efficiency (ROC^AUC^ = 0.847, *p* < 0.001, Table 3). Furthermore, description of the agreement between estimated and measured GFR were shown in Fig. 3. CKD-EPIcr-cys equation presented the best agreement with the measured GFR (95%CI [−34.87, 40.83]).

**Table 3:** Diagnostic values of the equations

Equation	R	ROC^AUC^	Sensitivity
7MDRD	0.681	0.816	0.72
aMDRD	0.672	0.802	0.71
re-aMDRD	0.672	0.802	0.71
CKD-EPIcr	0.696	0.816	0.72
CKD-EPIcys	0.675	0.833	0.73
CKD-EPIcr-cys	0.720	0.847	0.74

R: coefficient of relationship with measured GFR

ROC^AUC^: area under receiver operating characteristic curve

**Table 4:** Performance of the equations

Equation	Bias	Precision	Accuracy(%)		
			≤15 %	≤30 %	≤50 %
7MDRD	12.14**	23.33**	34.29**	57.55**	78.37**
aMDRD	14.77**	24.65**	29.80**	53.47**	74.69**
re-aMDRD	9.96**	23.33**	32.65**	60.41**	80.41**
CKD-EPIcr	13.51**	21.58**	30.61**	53.88**	78.78**
CKD-EPIcys	−4.24**	20.32**	38.78**	67.76	88.16
CKD-EPIcr-cys	2.98	19.31	44.49	69.39	87.35

Bias was the mean difference between estimated and measured GFR. Precision was the SD of this difference. Accuracy was the percentage of results deviating by ≤ 15, 30 and 50%from the meaured GFR. ** *P* < 0.01 versus the CKD-EPIcr-cys equation

### Performance of equations in subgroups

**Fig. 4:**
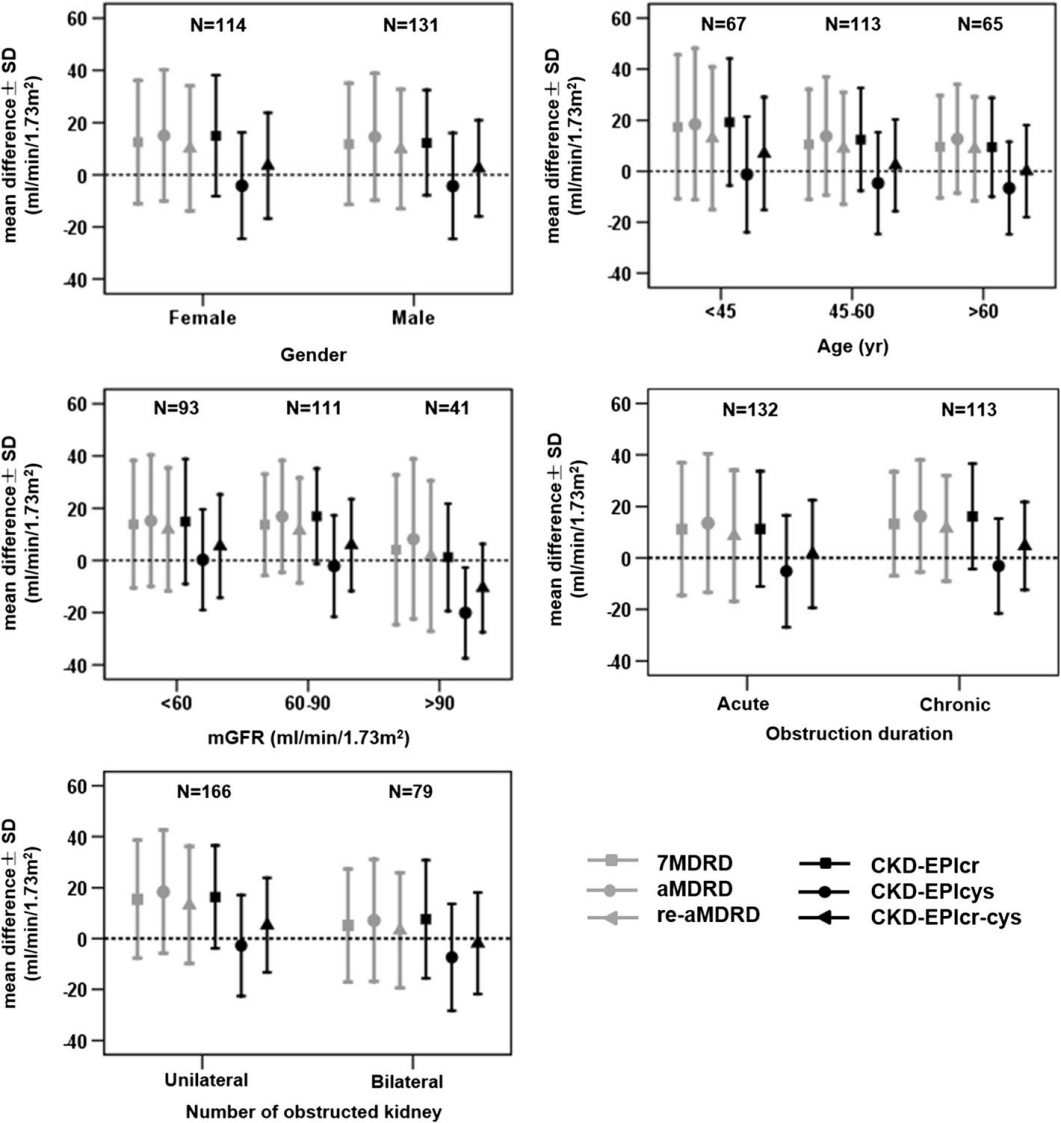
Comparison of the mean bias and precision over subgroups stratified by gender, age, and measured GFR. The mean bias was calculated as the mean of the differences between the estimated and measured GFR per subgroup, whereas the precision was the SD of this difference

In subgroups stratified by GFR, CKD-EPIcys equation exhibited the highest accuracy (52.69 %) and the smallest bias (0.27) in the group of the lowest GFR, while CKD-EPIcys equation and CKD-EPIcr-cys equation had an equally accuracy in patients with a GFR between 60 and 90 ml/min/1.73 m^2^. In the group of the highest GFR, CKD-EPIcr-cys equation remained the best, but both of them had larger bias in this group. For subgroups stratified by age, CKD-EPIcys equation had the best performance in the group of the youngest patients, with the highest accuracy (71.64 %) and the lowest bias (−1.24); in the other 2 age groups, CKD-EPIcr-cys equation still did the best. The bias of all the 6 equations was larger in women than in men. For both genders, the highest accuracy was reached by CKD-EPIcr-cys equation. Mean bias and precision for subgroups were presented in Fig. 4, and the values were shown in Table 5. In general, the 3 CKD-EPI equations provided better performance than the 3 MDRD equations, while CKD-EPIcr-cys equation gave the best correlation, the highest accuracy (*P* < 0.01) and diagnostic efficacy, and also the smallest bias (*P* < 0.01).

**Table 5:** Performance of the equations in subgroups

Variable	Subgroup	7MDRD			aMDRD			re-aMDRD			CKD-EPIcr			CKD-EPIcys			CKD-EPIcr-cys		
		B	P	A(30 %)	B	P	A(30 %)	B	P	A(30 %)	B	P	A(30 %)	B	P	A(30 %)	B	P	A(30 %)
GFR (ml/min/1.73 m^2^)	<60	13.83	24.39	40.86	15.20	25.13	39.78	11.82	23.57	43.01	14.88	23.90	37.63	0.27	19.33	52.69	5.49	19.83	51.61
	60–90	13.69	19.51	64.86	16.84	21.39	59.46	11.46	20.19	68.47	16.92	18.21	54.95	−2.15	19.45	76.58	5.90	17.64	76.58
	>90	4.08	28.73	75.61	8.18	30.63	68.29	1.68	28.91	78.05	1.19	20.60	87.80	−20.11	17.39	78.05	−10.62	16.94	90.24
Age (year)	<45	17.39	28.23	55.22	18.48	29.66	50.75	12.87	27.96	61.19	19.25	24.86	47.76	−1.24	22.72	71.64	6.92	22.09	64.18
	45–60	10.48	21.54	59.29	13.76	23.13	56.64	8.92	21.91	64.60	12.44	20.14	56.64	−4.66	19.96	72.57	2.31	18.00	75.22
	>60	9.61	20.04	12.70	12.70	21.28	50.77	8.77	20.39	52.31	9.45	19.39	55.38	−6.60	18.16	55.38	0.07	18.05	64.62
Gender	Male	11.80	23.19	64.12	14.53	24.31	56.49	9.83	22.85	64.12	12.24	20.12	59.45	−4.30	20.33	67.18	2.49	18.47	73.28
	Female	12.52	23.59	50.00	15.05	25.13	50.00	10.11	23.96	56.14	14.97	23.15	47.37	−4.17	20.40	68.42	3.54	20.30	64.91
Number of obstructed kidney	Unilateral	15.47	23.16	58.43	18.4	24.19	54.82	13.17	23.01	63.86	16.32	20.21	57.23	−2.74	19.87	72.29	5.29	18.59	74.70
	Bilateral	5.14	22.24	55.7	7.15	23.99	50.63	3.22	22.66	53.16	7.61	23.27	46.84	−7.38	21.03	58.23	−1.88	20.00	58.23
Disease duration	≥3 months	11.19	25.75	60.61	13.52	26.83	54.55	8.62	25.48	65.91	16.13	20.43	47.79	−3.13	18.47	73.45	4.65	17.13	70.80
	<3 months	13.24	20.2	53.98	16.24	21.73	52.21	11.53	20.54	53.98	11.28	22.36	59.09	−5.19	21.81	62.88	1.55	20.96	68.18

Bias was calculated as the mean difference between estimated and measured GFR. Precision was the SD of this difference. Accuracy (≤30 %) was the percentage of results deviating by 30 % from the measured GFR

## Discussion

Periodic monitoring of kidney function by a convenient and accurate method is necessary for early diagnosis of renal disease, individualized treatment and prognosis evaluation in clinical practice [3]. This pragmatic study was performed to compare 6 relatively popular GFR estimation equations in 245 Chinese patients with obstructive nephropathy, in order to test the applicability of the equations to pure obstructive nephropathy. As we expected, all these 6 eGFR equations were validated to estimate glomerular filtration function in obstructive nephropathy patients, and CKD-EPIcr-cys equation was more positively correlated with the ^99^mTc-DTPA GFR and had higher accuracy, even if there is the difference of pathogenesis, pathological and physiological changes and prognosis between obstructive nephropathy and diffuse renal diseases. Of all the 6 equations, the 3 CKD-EPI equations are generally more accurate than the 3 MDRD equations. Among the 3 MDRD equations, aMDRD equation is simpler to use than 7MDRD equation because it does not require inclusion of serum urea nitrogen and albumin concentration. Exclusion of these variables may also make the equation less susceptible to conditions in which serum urea nitrogen or albumin is strongly influenced by factors other than GFR. However, in our study, aMDRD equation did not perform better than 7MDRD equation. It was probably because of the difference in the serum creatinine measuring assay, which was assayed by the kinetic alkaline picrate in the original study of 7MDRD equation [6]. While in our study, it was measured by Roche enzymatic assay, which was more comparable to isotope dilution mass spectrometry (IDMS) -assigned values [8]. This may also explain the better performance of re-aMDRD equation, because re-aMDRD equation was developed by standardizing serum creatinine levels to an assay traceable to IDMS [8, 23]. The Kidney Disease Improving Global Outcomes (KDIGO) 2012 Clinical Practice Guideline for the Evaluation and Management of Chronic Kidney Disease recommended use of the 2009 CKD-EPI equation (CKD-EPIcr) instead of the MDRD study equation to estimate GFR from serum creatinine. And they suggest to use the 2012 CKD-EPI equations for GFR estimation (CKD-EPIcys, CKD-EPIcr-cys) [24]. It has already been validated that the 2012 CKD-EPI equations are applicable for Chinese population [25]. And our study results are also consistent with the guideline. There are several possible reasons for the best performance of the CKD-EPIcr-cys equation. Firstly, compared with MDRD equations, development and validation of the 3 CKD-EPI equations were conducted with large databases include participants with diverse clinical characteristics, with or without kidney disease, and across a wide range of measured GFR, thus allowing more general applicability than MDRD equations [9, 10]. Secondly, in the CKD-EPI equations, serum creatinine values are stratified according to gender and different cutoff values. However, in MDRD equations, there is no stratification. Thirdly, in comparison with MDRD, CKD-EPIcr-cys was expected to provide a more accurate estimated GFR, as cystatin C is a better glomerular filtration maker [26–28]. However, in our study, the CKD-EPI equation based on cystatin C alone was not more accurate than the creatinine estimates, suggesting that unmeasured and largely unknown non-GFR determinants of cystatin C are similar in magnitude to those of creatinine, which is consistent with the original publication [10]. Nevertheless, in subgroups stratified by age and gender, CKD-EPIcys equation exhibited higher accuracy than CKD-EPIcr equation. Thus, we did confirm the advantage of the cystatin C-based equation at less subject to the effects of age and gender than creatinine-based equations [8]. This study has its limitations. First, because of the retrospective data collection, most measurements of serum creatinine and GFR were not conducted on the same day. The daily changing creatinine and GFR may influence the results to some extent. Second, the measurement of serum cystatin C was not calibrated to standard method as did in the original study. Third, the sample size was relatively small, the validation of the 2012 CKD-EPI equations in pure obstructive nephropathy population can not represent the whole CKD population.

## Conclusion

In conclusion, the 3 CKD-EPI equations performed better than the 3 MDRD equations in estimating GFR in Chinese obstructive nephropathy patients; while among the 3 CKD-EPI equations, the CKD-EPI equation based on combined creatinine-cystatin C provided the best estimation of GFR. Our data suggested that the CKD-EPI equation based on combined creatinine-cystatin C should be widely used in general clinical practice to assess kidney function for obstructive nephropathy patients in China.

## Abbreviations

^99^mTc-DTPA: ^99^mTc-diethylenetriamine penta-acetate
CKD: Chronic kidney disease
CKD-EPI: Chronic Kidney Disease Epidemiology Collaboration
EDTA: Ethylene diamine tetraacetic acid
eGFR: estimated glomerular filtration rate
GFR: Glomerular filtration rate
IDMS: Isotope dilution mass spectrometry
KIDIGO: Kidney disease improving global outcomes
MDRD: Modification of diet in renal disease
mGFR: measured GFR
ROC^AUC^: Receiver operating characteristic
